# Do emotion regulation strategies mediate the attachment–paranoia association? An experimental study of repeated attachment imagery priming and stress buffering

**DOI:** 10.1111/papt.12398

**Published:** 2022-05-16

**Authors:** Monica Sood, Katherine B. Carnelley, Katherine Newman‐Taylor

**Affiliations:** ^1^ School of Psychology University of Southampton Southampton UK

**Keywords:** attachment, emotion regulation, paranoia, psychosis, security priming, stress buffering

## Abstract

**Objectives:**

Paranoia describes unfounded interpersonal threat beliefs. Secure attachment imagery attenuates paranoia, but limited research examines mechanisms of change and no studies examine how secure imagery may be implemented most effectively in clinical practice. In this study, we tested: (a) the causal impact of secure, anxious, and avoidant attachment imagery on paranoia and anxiety, (b) whether emotion regulation strategies mediate these relationships, and (c) whether secure imagery buffers against social stress.

**Design:**

We utilized a longitudinal, experimental design.

**Method:**

A general population sample with high non‐clinical paranoia (*N* = 265) completed measures of paranoia, anxiety, and emotion regulation strategies. Participants were randomly allocated to secure, anxious, or avoidant conditions and repeated an imagery prime for four days prior to a social stress task.

**Results:**

Relative to anxious and avoidant imagery, secure imagery decreased state paranoia and anxiety. These associations were not mediated by state emotion regulation strategies, and secure imagery did not buffer against stress. Exploratory analyses on trait variables revealed that: (a) hyperactivating strategies mediated the association between attachment anxiety and paranoia, and (b) suppression mediated the association between attachment avoidance and paranoia.

**Conclusions:**

Secure attachment imagery reduces state paranoia and anxiety and could be incorporated into psychotherapies to attenuate clinical paranoia. Measurement of state emotion regulation was problematic. Attachment imagery does not buffer stress; further research is required to test whether secure imagery facilitates recovery from stress. Attachment style is likely to account for trait paranoia via attachment‐congruent emotion regulation strategies. Research is now needed to determine if these strategies can be targeted to alleviate paranoia in clinical populations.

**Practitioner points**

- In clinical practice, secure attachment imagery may be effective in reducing state paranoia and distress.
- Attachment styles influence the way that people typically regulate emotion which, in turn, impacts paranoia. Emotion regulation may therefore be an important therapeutic target to attenuate paranoia and associated distress.
- Secure attachment imagery does not buffer the impact of stress. This result requires replication in clinical groups, and future research should test whether secure imagery facilitates recovery *after* stress.

## BACKGROUND

Secure attachment imagery priming reduces paranoia in non‐clinical (Sood et al., 2021; Sood & Newman‐Taylor, 2020) and clinical (Pitfield et al., 2020) groups, but the mechanisms involved, and our understanding of how secure attachment imagery may be implemented most effectively in clinical practice, are only partially understood. The literature suggests that people with psychosis engage in attachment‐congruent emotion regulation (ER) patterns which negatively impact social functioning and exacerbate paranoia (Lincoln et al., 2018). However, there is limited evidence of the role of ER in the attachment–paranoia association (Sood et al., 2022), and no evidence of whether secure imagery is effective in reducing paranoia under conditions of social stress.

ER describes the ability to manage emotions by redirecting them in a desired direction (Koole, 2009). Some ER strategies are typically identified as ‘adaptive’ because they tend to positively impact functioning and increase positive affect, such as reappraisal (re‐evaluating events in a helpful manner), acceptance (having an open attitude toward events), and putting into perspective (reconsidering the seriousness of an event) (Garnefski & Kraaij, 2007). Other ER strategies are identified as ‘maladaptive’ because they tend to impair functioning and increase negative affect, such as rumination (repetitive rehearsal of distressing thoughts), suppression (inhibition of thoughts and emotions), catastrophization (perceiving a situation as significantly worse than it is), and self/other blaming. Adaptive ER is associated with mental wellbeing, whereas maladaptive ER is associated with psychopathology cross‐diagnostically (Aldao, 2012).

Attachment theory (Bowlby, 1969) offers a framework to conceptualize the development and use of ER strategies. Attachment is typically classified as secure (marked by enduring trust), anxious (fear of abandonment), or avoidant (fear of intimacy) (Ainsworth et al., 1978; Hazan & Shaver, 1987). Secure adults have learned through their interactions with available and responsive attachment figures that acknowledging and displaying distress will elicit positive responses from others, and that their own actions can reduce distress (Shaver & Mikulincer, 2002). They tend to be confident in their ability to manage distress, show control over negative emotions, tend not to catastrophize, and use adaptive ER strategies such as reappraisal (Meredith et al., 2006; Mikulincer & Shaver, 2019).

Attachment anxious and avoidant individuals typically develop alternative (secondary) distress regulation strategies due to inconsistent or rejecting attachment figures. Attachment anxious adults tend to *hyperactivate* the attachment system when distressed, focusing on negative cognitions and emotions. Hyperactivation corresponds to catastrophization and rumination (Caldwell & Shaver, 2012; Meredith et al., 2006). Attachment avoidant adults tend to *deactivate* the attachment system when distressed and inhibit negative cognitions and emotions. Deactivation corresponds to emotional suppression (Caldwell & Shaver, 2012; Garrison et al., 2014; Wei et al., 2005), which often exacerbates distress (the *rebound effect* [Wegner et al. 1987]). While external displays of distress are typically diminished/inhibited in avoidant individuals, internal distress (if not fully suppressed) might be identified with physiological assessments (e.g., Diamond et al., 2006).

Several studies evidence hyperactivating and deactivating strategies in people with psychosis (Livingstone et al., 2009; O'Driscoll et al., 2014). Kimhy et al. (2012) found that, relative to healthy controls, people with schizophrenia used less reappraisal and more suppression, leading to increased social dysfunction. Lincoln et al. (2015) found that relative to healthy controls, participants with psychosis reported reduced awareness, tolerability, acceptance, and modification of emotions, each of which predicted higher levels of stress.

Hyperactivating and deactivating ER strategies are also associated with paranoia in clinical, high‐risk, and non‐clinical groups. Lincoln et al. (2018) found that following social exclusion (using ‘Cyberball’ [Williams et al., 2000]), catastrophizing, ruminating, and self‐ and other‐blaming increased paranoia, whereas reappraisal, acceptance, and refocusing decreased paranoia in clinically high‐risk‐of‐psychosis participants. Nittel et al. (2018, 2019) found that suppression predicted higher levels of paranoia in people with psychosis and non‐clinical controls.

The evidence indicates that people with psychosis, and paranoia specifically, tend to use fewer adaptive ER strategies associated with secure attachment, and more maladaptive ER strategies associated with insecure attachment. This suggests that people with paranoia might be engaging in attachment‐congruent ER patterns that negatively impact their social functioning and exacerbate distress (Kimhy et al., 2012).

Only one study has examined the mediatory role of ER in the attachment–paranoia association. Ascone et al. (2020) demonstrated that trait hyperactivating ER strategies (i.e., rumination and catastrophization) mediate the relationship between global (dispositional) attachment‐anxiety and clinical paranoia, suggesting that attachment anxious individuals tend to ruminate and catastrophize in negative situations which, in turn, exacerbates paranoia. However, no study has examined whether suppression mediates the association between attachment avoidance and paranoia – examining this would help determine whether suppression increases paranoia among attachment avoidant individuals. Importantly, research in this area has relied on cross‐sectional designs and measured (rather than manipulated) variables which precludes causal and temporal inferences. Additionally, no studies have examined whether adaptive ER strategies (e.g., putting into perspective and reappraisal) mediate the association between secure attachment and (reduced) paranoia.

### Attachment imagery priming

Attachment priming is an established method of increasing the accessibility of secure (or insecure) working models and observing whether this influences subsequent cognitions and emotions (Baldwin et al., 1996). Secure attachment imagery involves asking a person to create a mental image of someone who makes them feel safe, loved, and comforted, which triggers activation of secure mental representations in memory to create a sense of interpersonal security (Baldwin et al., 1996). This ‘felt security’ is similar to that induced by the real presence of supportive attachment figures (Rowe & Carnelley, 2003). In practice, secure attachment imagery may be similar to some forms of compassionate imagery (Gilbert, 2005). Our focus in this paper is on testing predictions that arise from applying attachment theory to paranoia, including the impact of secure attachment priming – an inherently interpersonal imagery task that contrasts with fears of others' intentions.

Importantly, attachment styles can be primed regardless of global attachment style. For example, an attachment avoidant individual can be primed with a secure attachment style which temporarily increases accessibility of secure attachment‐relevant memories/working models (Baldwin et al., 1996; Rowe & Carnelley, 2003). While global attachment styles influence *general* attachment‐relevant thoughts, emotions, and behaviours, attachment priming facilitates a temporary/’state’ attachment style, which influences momentary attachment‐relevant thoughts, emotions, and behaviours, with emerging evidence that repeated priming may sustain these effects (Rowe et al., 2020).

The benefit of using attachment priming is that we can examine the *causal* impact of attachment. Several studies show that attachment imagery priming causes changes in paranoia, with secure imagery attenuating paranoia (e.g., Sood et al., 2021). However, no studies have examined the impact of secure attachment imagery on ER (a core component of attachment working models) in people with psychosis/paranoia. We would predict that attachment imagery priming leads to use of ER strategies that are congruent with the primed attachment style and, in turn, impact paranoia. An investigation of the mediatory role of ER in the attachment imagery–paranoia association will increase our understanding of *how* secure imagery operates and inform interventions for paranoia as these mechanisms can be targeted in therapy.

No studies have examined how secure attachment imagery might best be applied in clinical practice. Previous studies have examined the effects of security priming on stress and examined two hypotheses: the *buffering hypothesis* states that security priming buffers the impact of stress, and the *recovery hypothesis* states that security priming facilitates reductions in stress following a stressor (Selcuk et al., 2012). While Selcuk found no evidence to support that security priming buffers the impact of recalling a negative memory on negative affect/thinking, recent research shows that security priming attenuates paranoia (Sood et al., 2021) and, therefore, security priming might buffer paranoia under conditions of social stress. We sought to test this for the first time.

### Current study

**FIGURE 1:**
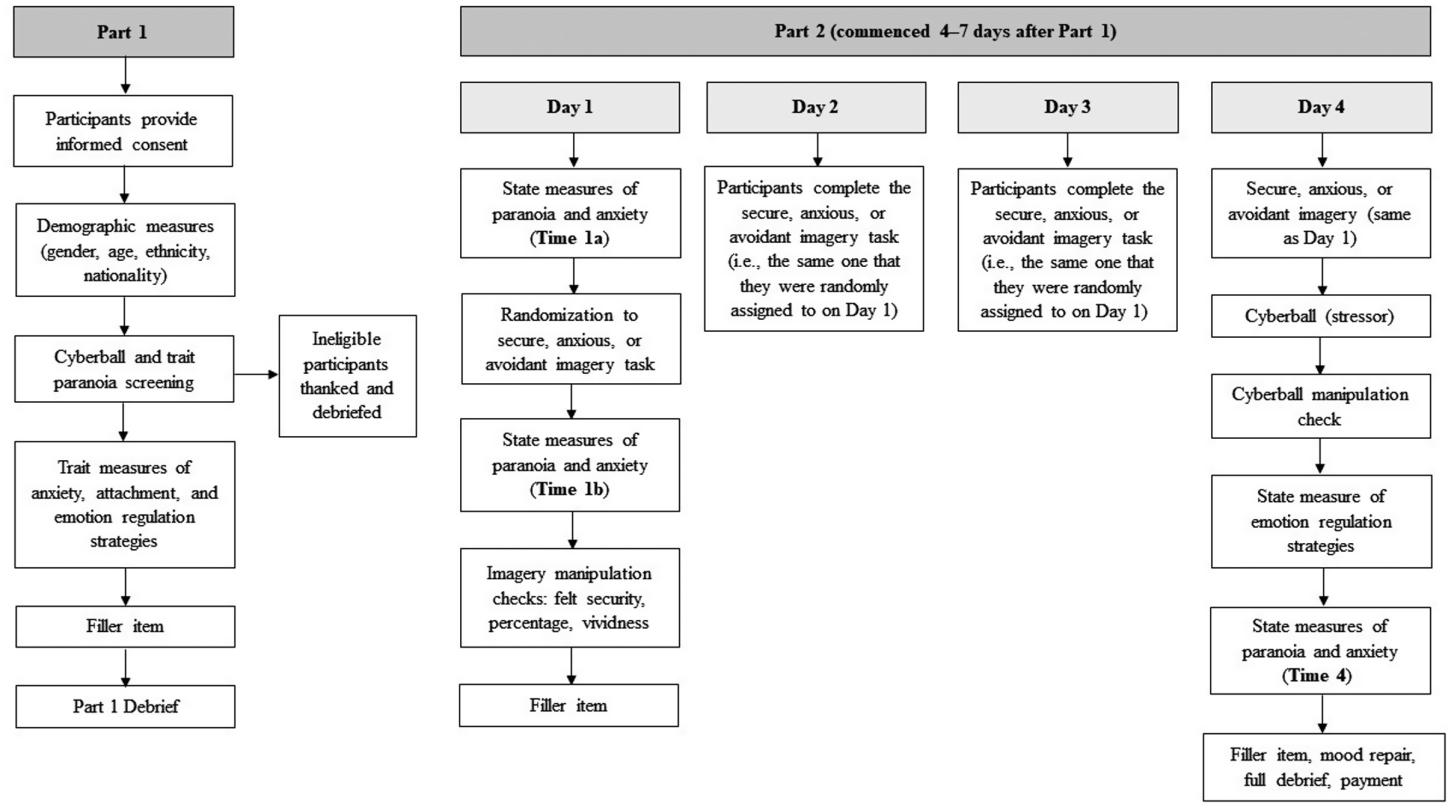
Study procedure*. Note*: Email address/Prolific ID was obtained from each participant at the beginning of Part 1 and all days of Part 2. Participants indicated if they experienced interruptions (and their nature) at the end of each day of Part 2

We sought to extend the emerging evidence base for secure attachment imagery as a therapeutic technique to attenuate paranoia. We examine the *causal* impact of attachment on paranoia using repeated attachment imagery (secure, anxious, and avoidant) and extend previous research by examining: (a) whether the impact of attachment imagery on paranoia is mediated by hyperactivating (rumination and catastrophization), deactivating (suppression and distraction^1^), and adaptive (reappraisal and putting into perspective) ER, and (b) whether secure imagery buffers against the impact of social stress, to increase our understanding of how secure imagery might best be applied when individuals are stressed. We expect that people primed with certain attachment styles will use ER strategies associated with those styles (e.g., primed avoidants should use more suppression). To assess this, in addition to baseline trait ER (how people generally regulate emotion), we assessed state ER (how people situationally regulate emotion following the prime). Given that paranoia (as a threat belief) is typically associated with anxiety, we included anxiety as a secondary outcome. See Table 1 for hypotheses, and Figure 1 for the study procedure and time‐points.

**TABLE 1:** Hypotheses

1.	Relative to anxious and avoidant imagery, secure imagery will reduce state paranoia and anxiety from pre‐imagery (Time 1a) to post‐imagery (Time 1b and 4).
2.	Impact of secure imagery and mediatory role of adaptive ER: Relative to the anxious and avoidant imagery groups, the secure imagery group will use less hyperactivating (rumination and catastrophization) and deactivating (suppression and distraction) ER, and more adaptive ER (reappraisal and putting into perspective), in response to a stressor (Cyberball), at Time 4. Adaptive ER will mediate the association between secure (vs. anxious and avoidant) attachment imagery and state paranoia and anxiety. Relative to the anxious and avoidant imagery groups, the secure imagery group will use more adaptive ER in response to stress and, thus, have lower levels of paranoia and anxiety.
3.	Impact of anxious imagery and mediatory role of hyperactivating ER: Relative to the avoidant imagery group, the anxious imagery group will use more hyperactivating (rumination and catastrophization) and less deactivating (suppression and distraction) ER in response to stress (Time 4). Hyperactivating ER will mediate the association between anxious (vs. secure and avoidant) attachment imagery and state paranoia and anxiety. Relative to the secure imagery group, the anxious imagery group will use more hyperactivating ER and, thus, have higher levels of paranoia and anxiety.
4.	Impact of avoidant imagery and mediatory role of deactivating ER: Relative to the anxious imagery group, the avoidant imagery group will use more deactivating (suppression and distraction) and less hyperactivating (rumination and catastrophization) ER in response to stress at Time 4. Deactivating ER will mediate the association between avoidant (vs. secure and anxious) attachment imagery and state paranoia and anxiety. Relative to the secure imagery group, the avoidant imagery group will use more deactivating ER strategies and, thus, have higher paranoia and anxiety.
5.	Buffering hypothesis: Relative to anxious and avoidant imagery, secure imagery will buffer the impact of stress on state paranoia and anxiety (i.e., reduce paranoia and anxiety post‐stressor).

*Note*: See Figure 1 for a summary of the procedure and time points.

## METHOD

The study was pre‐registered (23.03.21): https://osf.io/j8ckr

### Participants

We recruited adults from the general population with high levels of non‐clinical paranoia.^2^ Participants scoring **≥**53 (1*SD* above the original sample mean) on the Paranoia Scale were eligible (Bullock et al., 2016; Sood et al., 2021). Of 1110 people screened, 419 were eligible and 287 completed the study; the remaining participants dropped out. Mean replacement was used when <5% of data were missing for any participant on scale items; there were no cases with >5% missing data (Tabachnick & Fidell, 2013). One participant completed the study twice and 20 reported interruptions (one did not report) and thus were excluded from analyses. The final sample comprised 265 participants (112 females, 152 males, 1 non‐binary), aged 18–77 years (*M* = 24.86, *SD* = 8.25). Many were from Portugal (26%), Poland (23%), UK (10.2%), and Italy (9.1%). Most identified as White/British/Caucasian (67.9%).

The sample size was justified by an a priori G*Power analysis; for ANCOVA, to obtain .95 power and detect an effect of .25 at *p* = .05 with three groups and measurements, and one covariate, 251 participants are required. For mediation, Kline (2005) recommends 20 participants per parameter (we have >24).

### Measures

#### Paranoia

The Paranoia Scale (Fenigstein & Vanable, 1992) measures trait sub‐clinical paranoia. Participants rated 20 items (*α* = 0.62 for high‐paranoia sample; *α* = 0.91 for screened sample) from 1 (*not at all applicable to me*) to 5 (*extremely applicable to me*).^3^

The Adapted Paranoia Checklist (APC) measures state paranoia (Schlier et al. 2016). Participants rated five items from 0 (*not at all*) to 10 (*very much*). Internal consistency was good at Time 1a (*α* = 0.83), 1b (*α* = 0.88), and 4 (*α* = 0.83).

#### Anxiety

The State and Trait Anxiety Inventory (STAI) measures trait and state anxiety (Spielberger et al., 1983). Participants rated the frequency of 20   trait items (*α* = 0.90) from 1 (*almost never*) to 4 (*almost always*). We used a 6‐item version (STAI‐6) of the state subscale (Marteau & Bekker, 1992): Time 1a (*α* = 0.85), 1b (*α* = 0.92), and 4 (*α* = 0.85). Participants rated the frequency of state items from 1 (*not at all*) to 4 (*very much*).

#### Attachment style

The Experiences in Close Relationships Inventory‐Short‐Form (ECR‐12; Lafontaine et al., 2016) is a measure of trait attachment anxiety (*α* = 0.77) and attachment avoidance (*α* = 0.81) (6 items each). Participants rated items from 1 (*disagree strongly*) to 7 (*agree strongly*).

#### Emotion regulation

The Cognitive Emotion Regulation Questionnaire (CERQ)‐Brief (18 items; Garnefski et al., 2001) measures *trait* ER in response to negative events and comprises nine subscales: rumination (*α* = 0.71), catastrophization (*α* = 0.78), self‐blaming (*α* = 0.60), other‐blaming (*α* = 0.76), reappraisal (*α* = 0.67), putting into perspective (*α* = 0.64), acceptance (*α* = 0.74), positive refocusing (*α* = 0.72), and refocusing on planning (*α* = 0.73). In the absence of validated *state* suppression/distraction scales, and given that the CERQ does not measure suppression, we developed items to measure state and trait suppression (“I try to suppress my feelings”; “I try to keep difficult thoughts and feelings out of mind” [*α* = 0.67]) and distraction (“I try to distract myself” and “I try to turn my attention away” [*α* = 0.82]).^4^ Participants rated items from 1 (*almost never*) to 5 (*almost always*).

We also used the CERQ‐brief to measure *state* rumination (*α* = 0.79), catastrophization (*α* = 0.91), reappraisal (*α* = 0.79), and putting into perspective (*α* = 0.68). State suppression (*α* = 0.65) and distraction (*α* = 0.86) were measured using the items above. Participants reported how they were reacting *right now*, and rated items from 1 (*not at all*) to 5 (*completely*).

### Experimental manipulations

#### Attachment imagery

We used scripts to manipulate attachment imagery (Sood et al., 2021). These prompted participants to recall a time when they were with someone who made them feel safe and secure (secure imagery), worried that the person did not like or love them and wanted to pull away (anxious imagery), or uncomfortable when the person tried to get too close (avoidant imagery). Participants were asked to recreate the situation vividly, focusing on their senses.

#### Imagery manipulation‐checks

On Day 1, participants rated image‐vividness from 1 (*not at all*) to 10 (*very much*), percentage of time that the image was held in mind (0–100%), and felt security (Luke et al., 2012); that is, how comforted, secure, supported, safe, loved, and protected (*α* = 0.97) they felt from 1 (*not at all*) to 6 (*very much*).

### Stress‐induction

Cyberball (Williams et al., 2000) is a well‐established social‐stress paradigm in which participants play an online ball‐tossing game with two virtual players. The game elicits a sense of social exclusion and increases paranoia in non‐clinical (Stewart et al., 2017), clinical (Sundag et al., 2018), and clinically high‐risk for psychosis samples (Lincoln et al., 2018). Participants were told that they would play with two players and were instructed to imagine that they were playing the game in real life. Upon clicking “play”, participants saw a message saying “connecting to other players…” for five seconds. Subsequently, they saw three avatars on the screen labelled “player 1” (positioned top left), “player 2” (the participant; bottom middle), and “player 3” (top right). There were 24 ball tosses (max. 26 due to two depreciated throws). Participants were passed the ball twice at the beginning, after which they were excluded. They rated how good and bad they felt following the game from 1 (*not at all*) to 4 (*very much*).

### Procedure

The study had two parts, conducted online using Qualtrics (Figure 1). In Part 1, participants provided consent, demographics, and completed the Paranoia pre‐screen (Fenigstein & Vanable, 1992). They also indicated if they had ever heard of Cyberball and, if so, were ineligible. They then completed trait measures and a filler (to hide the study purpose and reduce demand characteristics). Participants with high paranoia were invited to Part 2, 4–7 days later, which lasted four days. They were asked to ensure that they were alone, without distractions. On each day, they listened to a 3‐minute audio‐recording that primed secure, anxious, or avoidant attachment imagery (participants were randomized using Excel simple randomization; they completed the imagery prime only once per day) and completed state measures of paranoia and anxiety on the first and last day. On the final day, participants also completed Cyberball and reported state ER. Participants indicated if they experienced interruptions (and their nature) at the end of the study daily.

## RESULTS

Between‐group differences on demographic and trait measures are reported in Tables S1–S3. Those with high levels of paranoia (*N* = 265) scored higher on attachment anxiety (*M* = 4.85, *SD* = 1.10) and avoidance (*M* = 4.14, *SD* = 1.20) than those who did not have high paranoia (*N* = 561): attachment anxiety (*M* = 3.99, *SD* = 1.19); avoidance (*M* = 3.38, *SD* = 1.26).

### Manipulation‐checks

Relative to the insecure imagery groups, the secure group reported higher felt‐security (*F*[2262] = 123.81, *p* < .001, η^2^ = 0.49). All groups reported comparable levels of image‐vividness (*F*[2262] = 0.52, *p* = .69). Participants, across all conditions, were able to hold the imagery in mind for 80% of the time, on average; however, between‐group differences emerged (*F*[2262] = 5.15, *p* = .006, η^2^ = 0.04) – the avoidant group held the image in mind for less time than the secure group (Table 2). All groups reported comparable levels of positive (*F*[2262] = 0.54, *p* = .22) and negative affect (*F*[2262] = 0.60, *p* = .21) post‐Cyberball and, as predicted, reported more negative than positive affect post‐Cyberball (Table 2).

**TABLE 2:** Descriptive statistics for demographics, trait measures, and manipulation‐checks

	Attachment imagery condition		
	Secure (*n* = 90)	Anxious (*n* = 86)	Avoidant (*n* = 89)
	*M* (*SD*)	*M* (*SD*)	*M* (*SD*)
Trait measures			
Age	24.54 (6.51)	25.45 (10.62)	24.62 (7.21)
Gender	–	–	–
Trait paranoia (PS)	63.36 (7.84)	65.14 (8.47)	63.12 (7.81)
Trait anxiety (STAI)	48.80 (4.97)	49.99 (4.94)	49.97 (4.88)
Trait rumination (CERQ)	6.66 (2.08)	7.09 (1.69)	6.80 (1.69)
Trait catastrophization (CERQ)	5.88 (2.13)	6.27 (2.20)	6.17 (2.01)
Trait suppression	5.81 (2.14)	6.53 (1.96)	6.16 (1.82)
Trait distraction	6.11 (2.24)	6.42 (1.96)	6.60 (2.17)
Trait positive reappraisal (CERQ)	6.57 (2.19)	6.92 (1.74)	6.27 (1.91)
Trait putting into perspective (CERQ)	5.70 (1.98)	5.91 (2.11)	5.67 (2.05)
Attachment anxiety (ECR)	4.45 (1.10)	4.77 (1.14)	4.93 (1.07)
Attachment avoidance (ECR)	4.14 (0.95)	4.15 (0.91)	4.27 (0.93)
Imagery manipulation checks			
Felt security	30.23 (5.35)	16.01 (8.78)	14.61 (7.55)
Vividness of image	7.24 (1.72)	7.06 (2.04)	6.94 (2.18)
Percentage image held in mind	8.14 (1.66)	8.20 (1.69)	7.39 (2.20)
Cyberball manipulation checks			
Positive affect post‐Cyberball	1.72 (0.71)	1.91 (0.75)	1.76 (0.75)
Negative affect post‐Cyberball	2.74 (0.92)	2.60 (1.03)	2.86 (0.89)

Abbreviations: CERQ, Cognitive Emotion Regulation Questionnaire; ECR, Experiences in Close Relationships Inventory; PS, Paranoia Scale; STAI, State–Trait Anxiety Inventory.

### ANCOVA: Impact of imagery on paranoia and anxiety

Mixed‐model Analyses of Covariance (ANCOVA) were conducted on state paranoia and anxiety with one between‐subjects factor (3 levels, attachment imagery: secure, anxious, and avoidant), one within‐subjects factor (3 levels, Time: 1a (pre‐imagery), 1b (post‐imagery), and 4 (post‐imagery+Cyberball), and one covariate (percentage of time image held in mind, given the between‐group differences on this variable) (Hypotheses 1 and 4). Simple effects tests with multiple comparisons and paired *t*‐tests were conducted to explore between‐ and within‐group differences. To reduce the impact of multiple‐testing, we applied a conservative *p*‐value of 0.01 to all analyses. There were no univariate outliers (*z* > ±3.29) on most variables.^5^ No variables were skewed; state anxiety (Time 1b) was slightly platykurtic.^6^

**FIGURE 2:**
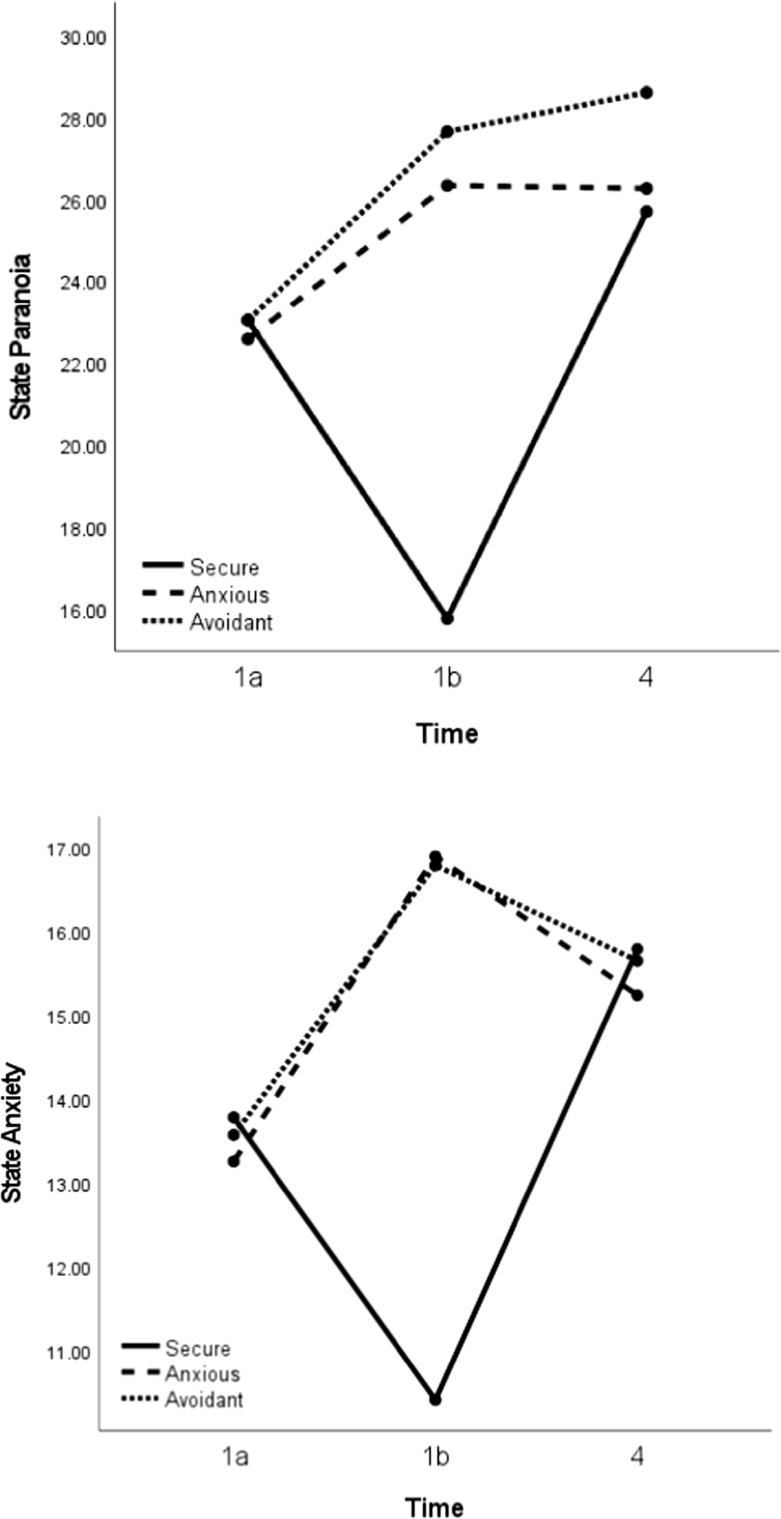
Change in state paranoia and anxiety over time in the secure, anxious, and avoidant attachment imagery conditions. *Note*: See Figure 1 for a summary of the procedure and time points. Covariates appearing in the model are evaluated at the following values: Percentage image held in mind = 7.91

Descriptive statistics for the state measures are reported in Table 3. There were main effects of imagery condition, but not time, and condition×time interactions for both paranoia and anxiety (Table 4, Figure 2).

**TABLE 3:** Descriptive statistics for state measures in the secure, anxious, and avoidant imagery conditions

	Secure imagery (*n* = 90)			Anxious imagery (*n* = 86)			Avoidant imagery (*n* = 89)		
	Time 1a	Time 1b	Time 4	Time 1a	Time 1b	Time 4	Time 1a	Time 1b	Time 4
	*M* (*SD*)	*M* (*SD*)	*M* (*SD*)	*M* (*SD*)	*M* (*SD*)	*M* (*SD*)	*M* (*SD*)	*M* (*SD*)	*M* (*SD*)
Paranoia (APC)	22.91 (8.59)	15.59 (8.03)	25.86 (9.19)	22.70 (9.56)	26.50 (10.10)	26.06 (9.58)	23.03 (8.59)	27.63 (9.47)	28.58 (9.55)
Anxiety (STAI‐6)	13.64 (4.55)	10.34 (3.47)	15.81 (3.98)	13.38 (4.15)	16.94 (4.69)	15.20 (4.29)	13.56 (3.94)	16.78 (4.27)	15.64 (4.16)

	Secure imagery – Time 4	Anxious imagery – Time 4	Avoidant imagery – Time 4
Rumination (CERQ)	5.62 (2.05)	5.38 (2.02)	5.88 (1.91)
Catastrophization (CERQ)	4.71 (2.39)	4.45 (2.31)	5.06 (2.46)
Suppression	5.50 (2.06)	4.94 (2.06)	5.12 (1.71)
Distraction	5.64 (2.39)	4.94 (2.17)	5.46 (2.38)
Positive reappraisal (CERQ)	5.17 (2.14)	5.41 (2.23)	5.11 (2.16)
Putting into perspective (CERQ)	7.09 (2.08)	7.56 (2.13)	7.16 (2.10)

*Note*: Time 1a = pre‐imagery, day 1; Time 1b = post‐imagery, day 1; Time 4 = post‐imagery+Cyberball, day 4; see Figure 1 for a summary of the procedure and time points.

Abbreviations: APC, Adapted Paranoia Checklist; CERQ, Cognitive Emotion Regulation Questionnaire; STAI‐6, State Anxiety Inventory‐Brief.

**TABLE 4:** Analyses of variance, simple effects, and post hoc paired *t*‐test statistics

	*F*	*p*	Effect size
Paranoia (df)			
Imagery condition (2, 261)	13.66	<.001	0.09
Time (2, 261)	0.59	.55	**–**
Imagery condition × Time (4, 261)	18.60	<.001	0.13
Simple effects T1a (2, 262)	0.03	.97	**–**
Simple effects T1b (2, 262)	46.36	<.001	0.26
Simple effects T4 (2, 262)	2.30	0.10	**–**
Percentage (1, 260)	0.11	.74	**–**
Percentage × Time (2, 261)	0.45	.64	**–**
Anxiety (df)			
Imagery condition (2, 261)	14.58	<.001	0.10
Time (2, 261)	1.56	.21	**–**
Imagery condition × Time (4, 261)	33.68	<.001	0.21
Simple effects T1a (2, 262)	0.09	.92	**–**
Simple effects T1b (2, 262)	72.67	<.001	0.36
Simple effects T4 (2, 262)	0.51	.60	**–**
Percentage (1, 260)	0.61	.44	**–**
Percentage × Time (2, 261)	1.17	.31	**–**

	Secure imagery					Anxious imagery					Avoidant imagery				
	*t*(89)	Lower 95% CI	Upper 95% CI	*p*	*d*	*t*(85)	Lower 95% CI	Upper 95% CI	*p*	*d*	*t*(88)	Lower 95% CI	Upper 95% CI	*p*	*d*
Change in Paranoia															
T1a to T1b	13.41	6.24	8.41	<.001	0.88	−4.04	−5.66	−1.93	<.001	0.39	−5.28	−6.33	−2.86	<.001	0.51
T1b to T4	−8.07	−12.80	−7.74	<.001	1.19	−8.17	−2.39	3.28	<.001	**–**	−0.69	−3.71	1.80	.49	**–**
Change in Anxiety															
T1a to T1b	8.98	2.57	4.03	<.001	0.82	−7.27	−4.53	−2.58	<.001	0.80	−7.60	−4.05	−2.37	<.001	0.78
T1b to T4	−9.61	−6.60	−4.34	<.001	1.47	2.68	3.04	2.68	.01	0.39	1.88	−0.07	1.88	.06	**–**

*Note*: T1a = Time 1 (pre‐imagery, day 1); T1b = Time 2 (post‐imagery, day 1); T4 = Time 4 (post‐imagery+Cyberball, day 4); see Figure 1 for a summary of the procedure and time points. Text in parenthesis are degrees of freedom. Partial eta squared (η_p_^2^) is reported for main effects and interactions, eta squared (η^2^) for simple effects tests, and *d* (*M*_2_‐*M*_1_) / *SD*_pooled_) for paired *t*‐tests.

#### Between‐group differences

As predicted, the three conditions did not differ in paranoia and anxiety at Time 1a (pre‐imagery), but differed at Time 1b (post‐imagery). At Time 1b, relative to the secure imagery condition, the anxious and avoidant imagery conditions reported higher paranoia and anxiety, as hypothesized (Table 3). Contrary to hypotheses, the groups did not differ in paranoia and anxiety at Time 4 (post‐imagery+Cyberball).

#### Within‐group differences

From Time 1a to 1b (pre‐ to post‐imagery), paranoia and anxiety decreased in the secure imagery condition and increased in the anxious and avoidant imagery conditions, as hypothesized (Table 4). In the secure imagery group, higher levels of paranoia and anxiety were observed at Time 4 (post‐stressor) than Time 1b (post‐imagery). In the anxious and avoidant imagery groups, there were no differences in paranoia levels between Time 1b and Time 4. In the anxious imagery group, anxiety was lower at Time 4 than 1b, and did not differ at these time points for those in the avoidant imagery group.

There was no main effect of percentage image held in mind nor a percentage×time interaction for both paranoia and anxiety.

### ANOVA: Impact of imagery on ER

One‐way ANOVAs were used to test Hypotheses 2a, 3a, and 4a. Counter to expectations, there were no between‐imagery group differences on any of the state ER strategies (see Table S1 in the supplementary material).

### Mediation: Role of ER in the attachment imagery–paranoia association

We tested parallel mediation using PROCESS version 3 (Hayes, 2018) to examine whether hyperactivating (rumination and catastrophization), deactivating (suppression and distraction), and adaptive ER strategies (reappraisal and putting into perspective) mediated the association between attachment imagery and paranoia and anxiety.

Dummy coding was used to produce two variables: anxious relative to secure imagery (*D*_1_), and avoidant relative to secure imagery (*D*_2_) (Hayes & Preacher, 2014). Because we expected differences between the anxious and avoidant groups, we reconducted the analyses with the anxious imagery condition as the reference category (i.e., secure relative to anxious imagery [*D*_3_] and avoidant relative to anxious imagery [*D*_4_]; only results for *D*_4_ are reported). *Relative specific* indirect effects^7^ were inferred using percentile bootstrap confidence intervals (CI) with 5000 bootstrapped samples. Mediation is observed when the 95% CIs do not cross zero (Hayes, 2018). We report partially standardized indirect effects (*ab*_ps_; Hayes, 2018) and infer these following Kenny's (2018) designation of small (.01), medium (.09), and large (.25).

Rumination and catastrophization, and suppression and distraction, were positively correlated (Table 5) and thus combined into hyperactivating (*α* = 0.84) and deactivating (*α* = 0.81) variables, respectively. Putting into perspective was not correlated with reappraisal, deactivating ER, or paranoia, and therefore was dropped from mediation analyses. There were no correlations between reappraisal and anxiety, so we did not examine reappraisal in the anxiety model.

**TABLE 5:** Correlation matrix for state variables at time 4 (post‐imagery + Cyberball)

Scale	1	2	3	4	5	6	7	8	9
(1) Paranoia	–								
(2) Anxiety	.54**	–							
(3) Rumination	.46**	.50**	–						
(4) Catastrophization	.53**	.53**	.56**	–					
(5) Suppression	.26**	.18**	.26**	.30**	–				
(6) Distraction	.28**	.24**	.18**	.27**	.56**	–			
(7) Reappraisal	.16**	.05	.27**	.20**	.30**	.19**	–		
(8) Putting into Perspective	−.08	−.20**	−.07	−.14*	.02	.09	.09	–	
(9) Hyperactivating ER	.01	−.05	.05	−.02	.02	.00	.02	.11	–
(10) Deactivating ER	−.09	−.03	−.10	−.01	−.06	.04	.03	−.06	.11

*Note*: Hyperactivating ER = rumination and catastrophization combined. Deactivating ER = suppression and distraction combined. See Figure 1 for a summary of the procedure and time points.

* *p* < .05. ** *p* < .01.

**FIGURE 3:**
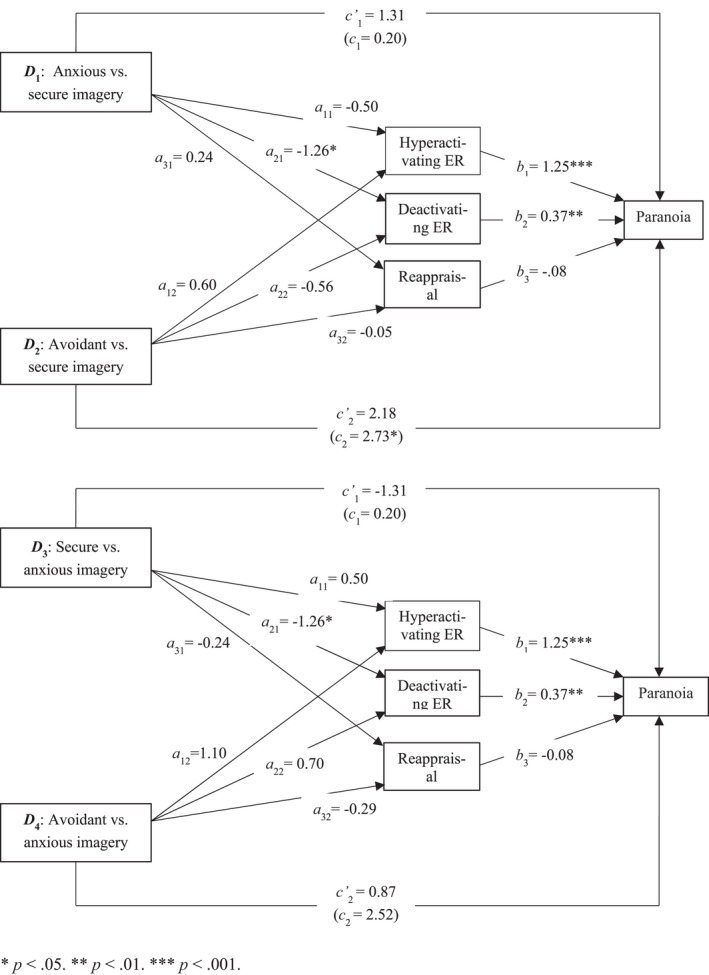
Mediation of the effect of attachment imagery (anxious and avoidant vs. secure – upper panel; secure and avoidant vs. anxious – lower panel) on paranoia by emotion regulation

We first tested whether hyperactivating ER, deactivating ER, and reappraisal mediated the imagery–paranoia association (Figure 3). There was no direct effect of *D*_1_, but there was a direct effect of *D*_2_, on state paranoia (Table 6). There were no indirect effects for *D*_1_ or *D*_2_ through hyperactivating ER and reappraisal. There was an indirect effect for *D*_1,_ but not *D*_2,_ on paranoia through deactivating ER. Relative to the secure imagery condition, the anxious imagery condition used less deactivating ER in response to stress and thus had higher paranoia, though this effect was small (*a*_21_ *b*_2ps_ = −0.05, *SE* = 0.03, 95% CI = [−0.13, −0.001]). The same model was tested to compare anxious and avoidant imagery (*D*_4_; Figure 3), however, no direct or indirect effects emerged (Table 6).

**TABLE 6:** Relative direct and indirect effects of attachment imagery on state paranoia and anxiety via emotion regulation

	Path coefficient	Standard error	Lower 95% CI	Upper 95% CI	*p*
State Paranoia					
Direct effect of *D*_1_	1.31	1.18	–	–	.27
Direct effect of *D*_2_	2.18	1.17	–	–	.06
Direct effect of *D*_4_	0.87	1.19	–	–	.47
Indirect effects of *D*_1_					
Hyperactivating ER	−0.62	0.73	−2.06	0.84	–
Deactivating ER	−0.47	0.31	−1.20	−0.01	–
Reappraisal	−0.02	0.12	−0.33	0.17	–
Indirect effects of *D*_2_					
Hyperactivating ER	0.75	0.73	−0.66	2.20	–
Deactivating ER	−0.21	0.24	−0.76	0.22	–
Reappraisal	0.00	0.09	−0.23	0.18	–
Indirect effects of *D*_4_					
Hyperactivating ER	1.37	0.75	−0.07	2.93	–
Deactivating ER	0.26	0.25	−0.13	0.86	–
Reappraisal	0.02	0.13	−0.22	0.33	–
State anxiety					
Direct effect of *D*_1_	−0.24	0.51	–	–	.65
Direct effect of *D*_2_	−0.50	0.51	–	–	.32
Direct effect of *D*_4_	−0.26	0.51	–	–	.61
Indirect effects of *D*_1_					
Hyperactivating ER	−0.30	0.35	−0.98	0.42	–
Deactivating ER	0.36	0.35	−0.33	1.06	–
Indirect effects of *D*_2_					
Hyperactivating ER	−0.08	0.09	−0.27	0.08	–
Deactivating ER	−0.03	0.06	−0.17	0.07	–
Indirect effects of *D*_4_					
Hyperactivating ER	0.66	0.36	−0.02	1.38	–
Deactivating ER	0.04	0.06	−0.06	0.20	–

*Note*: *D*_1_ = anxious relative to secure attachment imagery. *D*_2_ = avoidant relative to secure attachment imagery. *D*_4_ = avoidant relative to anxious attachment imagery. Estimated path coefficients are unstandardized.

**FIGURE 4:**
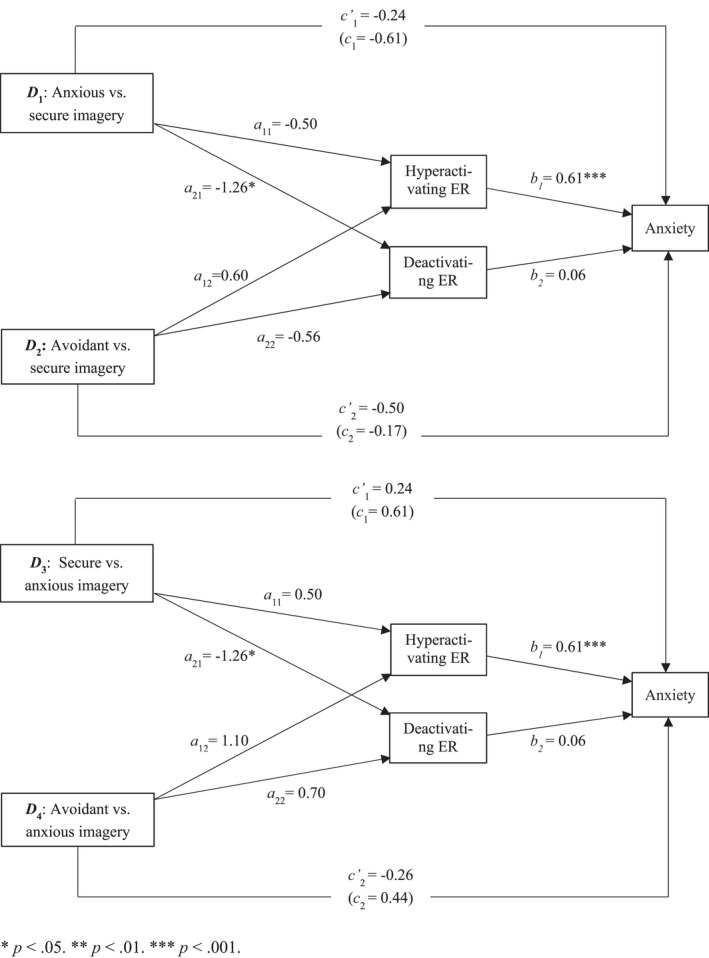
Mediation of the effect of attachment imagery (anxious and avoidant vs. secure – upper panel; secure and avoidant vs. anxious – lower panel) on anxiety by emotion regulation

We next tested whether hyperactivating and deactivating ER mediated the imagery–anxiety association (Figure 4). No direct or indirect effects emerged for *D*_1_, *D*_2_, or *D*_4._ 8

The mediation results contradict our hypotheses and suggest that there are problems with our measurement of state ER (we elaborate this argument in the discussion). Previous research shows that trait hyperactivating ER strategies mediate the association between global attachment anxiety and trait paranoia (Ascone et al., 2020); however, no studies have examined whether trait deactivating ER strategies (i.e., suppression) mediate the association between global attachment avoidance and trait paranoia, constituting a gap in the literature (Sood et al., 2022). For this reason, and given the issues with our state measure of ER, we conducted exploratory mediation analyses to replicate Ascone et al.’s (2020) findings in a non‐clinical group and, for the first time, investigate the role of emotional suppression in the attachment–paranoia association.

**FIGURE 5:**
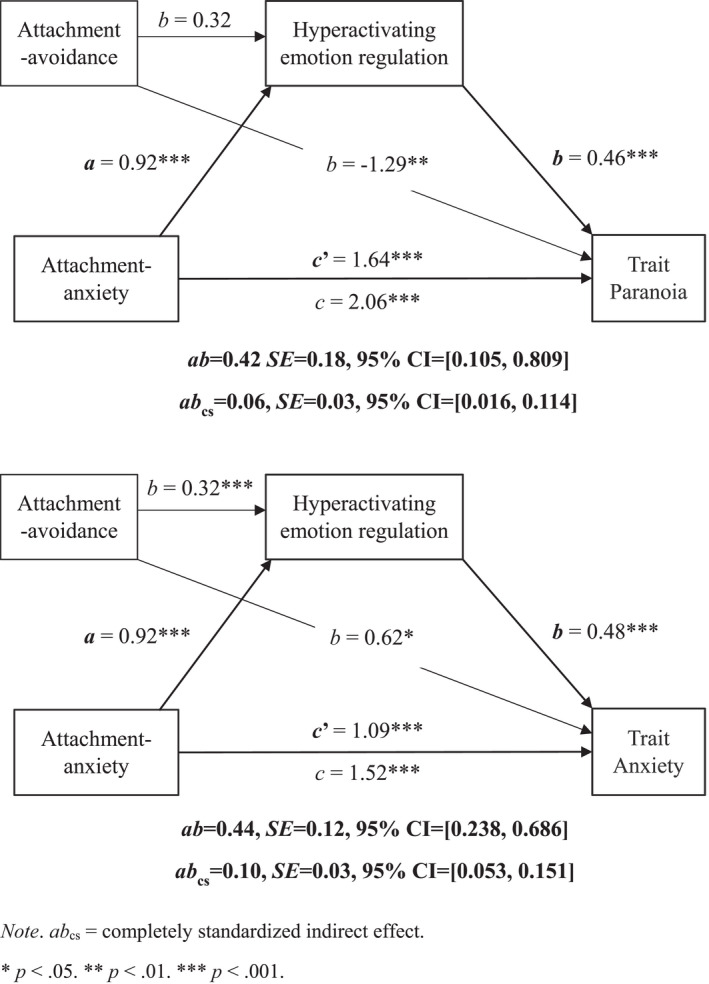
Mediation of the effect of global attachment anxiety on trait paranoia (upper panel) and anxiety (lower panel) by hyperactivating emotion regulation, holding global attachment avoidance constant

Trait catastrophizing and rumination were combined into a ‘hyperactivating ER’ variable (*α* = 0.73). Attachment avoidance was correlated with trait suppression but not distraction, and distraction was not associated with trait paranoia or anxiety (Tables S1–S3); we therefore dropped distraction from exploratory analyses. We examined whether: (a) trait hyperactivating ER mediates the association between attachment anxiety and trait paranoia and anxiety, holding attachment avoidance constant and (b) trait suppression mediates the association between attachment avoidance and trait paranoia and anxiety, holding attachment anxiety constant.^9^ There were indirect effects of global attachment anxiety on both trait paranoia and anxiety via hyperactivating ER. Individuals high in attachment anxiety used more hyperactivating ER which, in turn, was associated with higher trait paranoia and anxiety (Figure 5).

**FIGURE 6:**
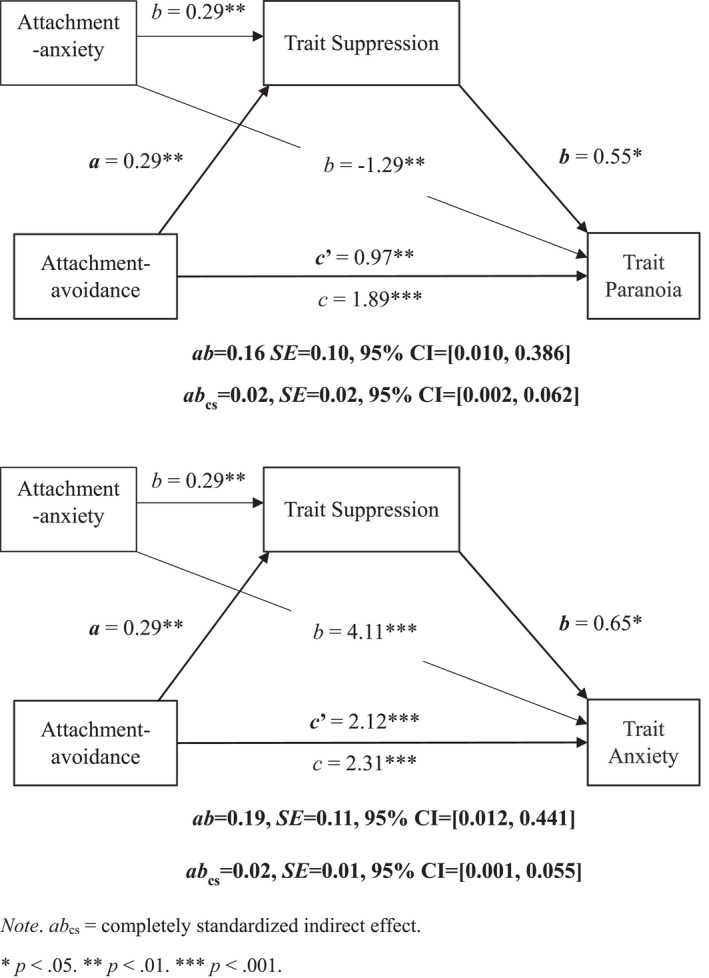
Mediation of the effect of global attachment avoidance on trait paranoia (upper panel) and anxiety (lower panel) by trait suppression, holding global attachment anxiety constant

There were also indirect effects of global attachment avoidance on trait paranoia and anxiety via suppression. Higher attachment avoidance was associated with greater use of suppression which, in turn, was associated with higher levels of paranoia and anxiety (Figure 6).

## DISCUSSION

We sought to demonstrate the relationship between attachment imagery and paranoia and anxiety, and determine whether these associations are mediated by hyperactivating, deactivating, and adaptive ER. We also investigated whether secure imagery buffers against the impact of social stress on paranoia and anxiety. As predicted, there were immediate decreases in state paranoia and anxiety following secure imagery, and increases following insecure imagery. However, contrary to hypotheses, the imagery–paranoia and imagery–anxiety associations were not mediated by state ER, and secure imagery did not buffer against stress.

### Impact of imagery on paranoia

In line with previous research (Bullock et al., 2016; Newman‐Taylor et al., 2017; Newman‐Taylor et al., 2021; Sood et al., 2021; Sood & Newman‐Taylor, 2020), the results demonstrate that, relative to anxious and avoidant imagery, secure‐attachment imagery effectively reduces state paranoia and anxiety. Manipulation of attachment using priming demonstrates that the effects observed are causal. This has now been demonstrated consistently in non‐clinical experiments but requires replication with clinical groups.

### Buffering hypothesis

Having established that secure‐attachment imagery effectively reduces paranoia, we sought to investigate how it might be applied clinically. Specifically, we examined whether secure imagery buffers against the impact of social stress. We predicted that, relative to anxious and avoidant imagery, secure imagery would decrease paranoia and anxiety after participants are exposed to stress. However, there were no between‐group differences in paranoia and anxiety post‐stressor and, in the secure imagery group, state paranoia and anxiety were significantly higher post‐stressor (Time 4) than post‐imagery (Time 1b), suggesting that secure imagery failed to buffer the impact of the stressor. While it is possible that the repeated imagery was less effective at Time 4 due to practice effects, a recent systematic review suggests that repeated attachment primes tend to have a cumulative effect (Rowe et al., 2020). Given the elevated levels of attachment insecurity in the high‐paranoia group, it may take more time and further practice of the secure imagery task to sustain felt security over time.

Previous research has also failed to support the buffering hypothesis. Hutton et al. (2017) asked participants to complete imagery tasks (secure attachment, positive affect, or neutral), and then induced paranoia (participants completed an unsolvable task while being filmed). They found that secure attachment priming did not buffer paranoid thinking after the task. The study was limited by its small student sample and lack of manipulation‐checks, making it difficult to ascertain whether the primes induced felt security. Selcuk et al. (2012) found that activating mental representations of attachment figures before stress‐induction (recalling a negative memory) did not reduce negative affect or thinking, but activating these representations *after* stress‐induction did. Similarly, Ai et al. (2020) found that salivary cortisol concentrations increased when security priming was administered before the stressor, and decreased when administered after. This suggests that secure imagery may be more helpful in *facilitating recovery from*, rather than buffering, stress. Research is needed to test the recovery hypothesis directly, examining whether secure attachment imagery facilitates reductions in paranoia post‐stress, and the impact on ER.

### Impact of imagery on ER

The imagery tasks did not affect state ER strategies. One explanation for this surprising finding is that, in moments of stress, participants use multiple ER strategies. For example, people may begin to catastrophize, then tell themselves ‘it's not a big deal’ or ‘there are worse things’, and later turn their attention away or distract themselves. This would explain unexpected positive correlations between hyperactivating, deactivating, and adaptive ER strategies in this study. Research suggests that the ability to shift flexibly between different ER strategies and do what is more appropriate to the context is the norm in healthy populations, but this flexibility is often absent in clinical groups (Kashdan & Rottenberg, 2010). Trait and state ER did not correlate with one another in this study, suggesting that trait ER does not determine the use of state ER strategies; this supports the notion that people, especially those in healthy populations, are able to regulate emotions flexibly, based on the situation/context. This suggests that our method of measuring state ER is problematic, and self‐report measures of state ER may be ineffective when researchers are interested in examining dominant or specific ER strategies that, for example, maintain distress, particularly with non‐clinical groups. Qualitative methods, such as think‐aloud protocols, may be preferred, allowing participants to describe, over time, how they are regulating emotion.

Another possibility is that the imagery tasks do not produce changes in explicit ER, but instead influence implicit ER – the process of changing the duration or intensity of emotional responses without conscious intention or control (Koole & Rothermund, 2011). If so, participants may have been regulating emotion at a subconscious level, but could not report this using the questionnaire. There is evidence that some ER strategies tend to be more automatic (e.g., rumination), whereas others tend to be more controlled (e.g., reappraisal) (Gross, 2007). It is possible that the primes are more effective in influencing automatic ER strategies; future research should test this possibility and utilize non‐questionnaire (e.g., psychophysiological) measures of affect (e.g., skin‐conductance or heart‐rate) to see how these correspond to self‐reported affect.

Furthermore, Cyberball may not have been an effective stressor in this study. The results showed that ‘putting into perspective’ had the highest mean in all conditions, suggesting that most participants agreed that there are worse things in life than their experiences in Cyberball. This suggests that a more distressing task may be required to elucidate imagery group differences in state ER. However, this explanation is implausible because: (a) Cyberball increased state paranoia and anxiety in the secure imagery group with large effects and maintained high paranoia and anxiety levels in the insecure imagery groups, (b) no indirect effects emerged when only those who were highly distressed following Cyberball were included in analyses, and (c) previous research consistently shows that Cyberball effectively increases paranoia (e.g., Lincoln et al., 2018). It is thus unlikely that Cyberball was ineffective, and the problem may therefore lie with the measurement of state ER.

### ER strategies as mediators in the imagery–paranoia association

Contrary to expectations, although hyperactivating and deactivating ER predicted heightened paranoia, and hyperactivating ER predicted increased anxiety, hyperactivating ER did not mediate the impact of anxious (vs. secure) imagery on paranoia or anxiety, and deactivating ER did not mediate the impact of avoidant (vs. secure) imagery on paranoia and anxiety. These results are likely to be due to problems with the measurement of ER described previously.

The results also showed that relative to the secure imagery group, the anxious imagery group used less deactivating ER and therefore had higher paranoia. A possible explanation is that some deactivating ER items could be interpreted in positive ways (e.g., the items “I turn my attention way” and “I distract myself” may not necessarily be maladaptive, and using distraction might be useful for those high in global attachment anxiety who are prone to ruminate). Additionally, it may be that distraction and suppression are adaptive in the context of Cyberball; for example, while avoidance is maladaptive for solvable problems, it is arguably optimal for unsolvable problems (Shaver & O'Connor, 1986). This might explain why the secure‐primed group used more avoidant ER and aligns with the broader ER literature which suggests that any ER strategy can be considered adaptive or maladaptive depending on the context and desired outcome (Gross, 2007). For example, Westermann et al. (2012) found that, in highly paranoia‐prone individuals, reappraisal (a typically adaptive ER strategy) increases paranoia under conditions of social exclusion (Cyberball).

Given the problems with measuring state ER, we conducted exploratory analyses with trait ER strategies as mediators in the trait attachment–paranoia association. These analyses showed that trait hyperactivating ER mediated the association between global attachment anxiety and trait paranoia and anxiety while controlling for global attachment‐avoidance. Individuals high in attachment‐anxiety used more hyperactivating ER which, in turn, was associated with higher trait paranoia and anxiety. These results align with those of Ascone et al. (2020), demonstrating reliability. This is the first study to show that trait suppression mediates the association between global attachment avoidance and trait paranoia and anxiety, suggesting that those high in global attachment avoidance use more suppression which, in turn, is associated with higher levels of paranoia and anxiety (albeit with a small effect). This aligns with research showing that attachment avoidant individuals tend to suppress attachment‐related needs and negative affect. Extending this literature, this study shows that suppression is associated with higher levels of paranoia and anxiety in attachment avoidant individuals with high non‐clinical paranoia ‐ demonstrating that these problems are present early.

### Strengths and limitations

We tested the causal impact of attachment on paranoia using a longitudinal, experimental design with repeated attachment imagery. The sample was fully powered, with a similar number of males and females and range of ethnicities and nationalities.

However, the psychometric properties of the state ER scale may have been flawed. There were only two items per subscale, which although face valid, may not have fully captured the constructs. The CERQ was designed to measure trait rather than state ER strategies, and the suppression and distraction items had not been assessed for validity and reliability. Future research should consider developing effective, standardized measures of *state* ER strategies. The study may also be limited by multiple testing, which inflates the risk of Type 1 error; to limit the impact of this, we used a conservative *p*‐value across analyses.

Our method of identifying a high‐paranoia group has been used in several other research studies (e.g., Combs & Penn, 2004; Bullock et al., 2016; Newman‐Taylor et al., 2021; Parker & Kingston, 2021; Sood et al., 2021), enabling cross‐study comparisons. However, comparisons are more difficult with studies that have used different paranoia assessments and cut‐offs. Research examining low and high paranoia thresholds across multiple instruments would be a valuable addition to the literature.

Although attachment imagery was manipulated, and we are confident that changes in state paranoia and anxiety are due to the imagery manipulation, the trait ER strategies were not manipulated and so we cannot ascertain whether these *cause* changes in paranoia and anxiety. Nevertheless, this is the first study to show the mediatory role of suppression in the attachment–paranoia association.

### Implications

Secure‐attachment imagery is an effective means of attenuating paranoia and anxiety in people with non‐clinical and clinical paranoia. Our method of measuring state ER was problematic, and we recommend that others do not use this approach. The role of trait ER in the attachment–paranoia association suggests that training individuals to use effective ER strategies (in addition to targeting unhelpful cognitions and behaviours) may be a useful addition to CBT to support clinical (symptom‐reduction) and recovery (quality‐of‐life) outcomes, which are currently modest (Jones et al., 2018). The selection of particular skills in ER training might be based on an individual's attachment style, for example, attachment avoidant individuals could be trained to use less suppression and more emotional expression, whereas attachment anxious individuals could be trained to use refocusing strategies to reduce the use of catastrophizing and ruminating.

The results also suggest that secure imagery does not buffer stress. Future research should test whether secure imagery facilitates recovery after stress – if so, psychotherapies might support people to use secure imagery when distressed or after discussing difficult topics such as trauma or adversity (which are common in clinical populations with psychosis [Varese et al., 2012]), to enable people to develop means of feeling safe when faced with perceived threat.

## AUTHOR CONTRIBUTIONS

**Monica Sood: Conceptualization; Data curation; Formal analysis; Funding acquisition; investigation; methodology; Project administration; visualization; Writing–original draft; Writing–review&editing. Katherine B. Carnelley: Conceptualization; methodology; supervision; Writing–review&editing. Katherine Newman‐Taylor: Conceptualization; methodology; supervision; Writing–review&editing.**

## CONFLICT OF INTEREST

None.

### OPEN RESEARCH BADGES

This article has a preregistered research design available at Open Science Framework

## Supporting information


Table S1
Table S2
Table S3

## Data Availability

The full dataset is available on the Open Science Framework Data Repository: https://osf.io/6awxn/. Full Citation: Sood, M., Carnelley, K., & Newman‐Taylor, K. (2021, September 29th). Does Emotion Regulation Mediate the Association between Attachment Imagery and Paranoia? Retrieved from osf.io/6awxn/.

## Footnotes

^1^
We included items measuring distraction given the conceptual overlap between suppression and distraction (i.e., both function as experiential avoidance and are likely to be used by people high in attachment avoidance). The choice of ER strategies was informed by adult attachment theory and evidence in psychosis groups.

^2^
Most participants (*n* = 257) were recruited from Prolific, a platform with a diverse participant pool on which people participate for financial compensation. The other participants were university students.

^3^
All alphas reported in the 'measures' section are for the current sample.

^4^
Limited *state* measures of suppression have been developed, and while established trait suppression subscales exist (e.g., Gross & John, 2003), many of these assess emotional expression (not showing emotions) rather than suppression (inhibiting emotions in an attempt to not feel them). Therefore, we sought to develop items to assess state emotional suppression, based on items that other authors have developed (e.g., Ehring et al., 2010; Nittel et al., 2018, 2019; Wegner & Zanakos, 1994), in addition to the original CERQ.

^5^
Outliers were observed on trait paranoia and anxiety. The results were the same with and without these outliers and, therefore, we included them in the final dataset. Examination of Cook's distances (which ranged from 0.06 to 0.09) suggests that the observations are unlikely to have unduly influenced the findings.

^6^
Field (2013) argues that ANOVAs and *t*‐tests are robust to minor departures from normality; we therefore continued analyses without changing the data.

^7^
These quantify the effect of *D*_1_ and *D*_2_ (or *D*_3_ and *D*_4_) on the outcome through each specific mediator.

^8^
The lack of mediation effects may be because not all participants found Cyberball stressful and, therefore, not all were regulating emotion. We therefore ran the mediation analyses on those who reported that they felt 'very bad' following Cyberball (*N* = 55). However, no effects emerged for either paranoia or anxiety, perhaps due to low statistical power. The mediation results were the same when ‘percentage image held in mind’ was controlled for.

^9^
People with both anxious and avoidant attachment styles typically regulate emotions in unhelpful ways. Given that both types of insecure attachment (anxious and avoidant) are associated with problematic responses to stress, attachment avoidance was controlled for in the attachment anxiety model, and vice versa, to ensure the results are specific to each attachment style. This is important so that therapies can be refined to target specific, attachment‐relevant ER strategies.
