# Supplementary material for: Do emotion regulation strategies mediate the attachment–paranoia association? An experimental study of repeated attachment imagery priming and stress buffering
Source: Psychol Psychother. 2022 May 16;95(3):781–806. doi: 10.1111/papt.12398 (PMC9543866; doi:10.1111/papt.12398)
Supplement: Supplementary file 1 — Table S1 Table S2 Table S3 [file PAPT-95-781-s001.docx]

**Table S1**

*Correlation Matrix for Trait and State Variables*

| Scale | 1 | 2 | 3 | 4 | 5 | 6 | 7 | 8 | 9 | 10 | 11 | 12 | 13 | 14 | 15 | 16 | 17 | 18 | 19 |
| --- | --- | --- | --- | --- | --- | --- | --- | --- | --- | --- | --- | --- | --- | --- | --- | --- | --- | --- | --- |
| **Trait Measures:** |  |  |  |  |  |  |  |  |  |  |  |  |  |  |  |  |  |  |  |
| (1) Paranoia | - |  |  |  |  |  |  |  |  |  |  |  |  |  |  |  |  |  |  |
| (2) Anxiety | .34** | - |  |  |  |  |  |  |  |  |  |  |  |  |  |  |  |  |  |
| (3) Rumination | .14* | .21** | - |  |  |  |  |  |  |  |  |  |  |  |  |  |  |  |  |
| (4) Catastrophization | .27** | .50** | .38** | - |  |  |  |  |  |  |  |  |  |  |  |  |  |  |  |
| (5) Suppression | .20** | .24** | .11 | .09 | - |  |  |  |  |  |  |  |  |  |  |  |  |  |  |
| (6) Distraction | .09 | .06 | .08 | .04 | .54** | - |  |  |  |  |  |  |  |  |  |  |  |  |  |
| (7) Reappraisal | .15* | -.38 | .22** | -.20** | -.09 | .00 | - |  |  |  |  |  |  |  |  |  |  |  |  |
| (8) Putting into perspective | -.01 | -.31** | .18** | -.18** | .10 | .12* | .42** | - |  |  |  |  |  |  |  |  |  |  |  |
| (9) Attachment anxiety | .27** | .45** | .19** | .34** | .14* | .11 | .19** | -.09 | - |  |  |  |  |  |  |  |  |  |  |
| (10) Attachment Avoidance | .14* | .23* | -.17** | -.04 | .15* | -.04 | -.24** | -.17* | -.12* | - |  |  |  |  |  |  |  |  |  |
| (11) Hyperactivating ER | .25** | .44** | .81** | .86** | .12 | .07 | -.01 | -.01 | .32** | -.12 | - |  |  |  |  |  |  |  |  |
| (12) Deactivating ER | .17** | .17** | .11 | .07 | .87** | .89** | -.05 | .13* | .14* | .06 | .11 | - |  |  |  |  |  |  |  |
| **Time 1a State Measures** |  |  |  |  |  |  |  |  |  |  |  |  |  |  |  |  |  |  |  |
| (13) Paranoia | .32** | .25** | .11 | .20** | .08 | .02 | -.20** | -.06 | .11 | -.14* | .19** | .05 | - |  |  |  |  |  |  |
| (14) Anxiety | .30** | .46** | .20 | .24** | .17** | .02 | -.31** | -.17** | .21** | -.18** | .21** | .11 | .41** |  |  |  |  |  |  |
| **Time 1b State Measures** |  |  |  |  |  |  |  |  |  |  |  |  |  |  |  |  |  |  |  |
| (15) Paranoia | .30** | .22** | .18* | .20 | .15* | .08 | -.10 | -.00 | .09 | .05 | .23** | .13* | .57** | .25** | - |  |  |  |  |
| (16) Anxiety | .25** | .34** | .17 | .25** | .14* | .06 | -.15* | -.08 | .13* | .03 | .25** | .11 | .19** | .42** | .67** |  |  |  |  |
| **Time 4 State Measures** |  |  |  |  |  |  |  |  |  |  |  |  |  |  |  |  |  |  |  |
| (17) Paranoia | .08 | .01 | -.06 | .07 | -.10 | -.06 | -.14* | -.06 | -.04 | .01 | .01 | -.09 | .02 | -.02 | .10 | .07 | - |  |  |
| (18) Anxiety | .07 | -.04 | -.05 | -.03 | -.05 | .00 | -.03 | -.03 | .04 | .04 | -.05 | -.03 | .02 | .02 | .08 | .02 | .54** | - |  |
| (19) Rumination | .06 | .08 | .03 | .05 | -.12 | -.06 | -.02 | -.02 | .08 | .04 | .05 | -.10 | .03 | -.06 | .06 | .00 | .46** | .50** | - |
| (20) Catastrophization | -.03 | -.03 | -.03 | -.00 | -.03 | .01 | -.08 | .01 | .05 | -.02 | -.02 | -.01 | -.05 | -.02 | .03 | -.01 | .53** | .53** | .56** |
| (21) Suppression | -.05 | .02 | -.05 | .07 | -.01 | .10 | .01 | .02 | .08 | -.08 | .02 | -.06 | .03 | .00 | -.03 | -.06 | .26** | .18** | .26** |
| (22) Distraction | -.03 | -.00 | -.05 | .05 | .04 | .04 | -08 | .10 | .03 | -.01 | .00 | .04 | .04 | .02 | .03 | -.06 | .28** | .24** | .18** |
| (23) Reappraisal | -.02 | .06 | -.03 | .06 | .03 | .02 | .04 | .02 | .11 | .02 | .02 | .03 | .07 | .07 | -.01 | .04 | .16** | .05 | .27** |
| (24) Putting into perspective | -.01 | .04 | .09 | .08 | -.03 | -.08 | .09 | .00 | -.07 | -.02 | .11 | -.06 | .04 | .05 | .07 | .09 | -.08 | -.20** | -.07 |
| (25) Hyperactivating ER | .01 | .03 | .00 | .03 | .22 | -.03 | -.06 | -.00 | .07 | .01 | .02 | .34 | -.02 | .21** | .23** | .25** | .01 | -.05 | .05 |
| (26) Deactivating ER | -.04 | .01 | .07 | .07 | .02 | -.03 | -.05 | .07 | .06 | -.05 | .01 | -.01 | .04 | .11 | .13* | .07 | -.09 | -.03 | -.10 |

Table S1 cont.

| Scale | 20 | 21 | 22 | 23 | 23 | 25 |
| --- | --- | --- | --- | --- | --- | --- |
|  |  |  |  |  |  |  |
| (20) Catastrophization | - |  |  |  |  |  |
| (21) Suppression | .30** | - |  |  |  |  |
| (22) Distraction | .27** | .56** | - |  |  |  |
| (23) Reappraisal | .20** | .30** | .19** | - |  |  |
| (24) Putting into perspective | -.14* | .02 | .09 | .09 | - |  |
| (25) Hyperactivating ER | -.02 | .02 | .00 | .02 | .11 | - |
| (26) Deactivating ER | -.01 | -.06 | .04 | .03 | -.06 | .11 |

* *p* < .05. ** *p* < .01.

**Between Group Differences at Baseline**

Between-group differences on demographic and trait measures (Table S2) were tested using one-way Analyses of Variance (ANOVA) and chi-square. There were no differences with one exception; the anxious-imagery group reported more suppression than the secure-imagery group.

**Table S2**

| Demographic/Trait Measure | *F*(2,262)  (*χ^2^(4, N=265)* | *p* | η^2^ |
| --- | --- | --- | --- |
| Age | 0.33 | 0.72 | - |
| Gender | 2.80 | *0.59* | *-* |
| Trait paranoia (PS) | 1.84 | 0.16 | - |
| Trait anxiety (STAI) | 1.69 | 0.19 | - |
| Trait rumination (CERQ) | 1.30 | 0.27 | - |
| Trait catastrophization (CERQ) | 0.81 | 0.44 | - |
| Trait suppression | 2.95 | 0.05 | 0.01 |
| Trait distraction | 1.19 | 0.31 | - |
| Trait positive reappraisal (CERQ) | 2.40 | 0.09 | - |
| Trait putting into perspective (CERQ) | 0.34 | 0.71 | - |
| Attachment-anxiety (ECR) | 0.47 | 0.63 | - |
| Attachment-avoidance (ECR) | 0.53 | 0.59 | - |

*Note*. PS = Paranoia Scale; STAI = State-Trait Anxiety Inventory; CERQ = Cognitive Emotion Regulation Questionnaire; ECR = Experiences in Close Relationships Inventory. Chi-squared (χ2) is reported for gender, and one-way ANOVA for all other variables.

**Table S3**

*Statistics for One-Way ANOVA on State Emotion Regulation Strategies*

|  | Descriptive statistics | | |  | ANOVA statistics | |
| --- | --- | --- | --- | --- | --- | --- |
|  | Secure | Anxious | Avoidant |  |  |  |
|  |  |  |  |  |  | |
|  | *M (SD)* | *M (SD)* | *M (SD)* |  | *F*(2,262) | *p* |
| Rumination | 5.62 (2.05) | 5.38 (2.02) | 5.88 (1.91) |  | 1.34 | 0.264 |
| Catastrophization | 4.71 (2.39) | 4.45 (2.31) | 5.06 (2.46) |  | 1.41 | 0.247 |
| Suppression | 5.50 (2.06) | 4.94 (2.06) | 5.12 (1.71) |  | 1.89 | 0.153 |
| Distraction | 5.64 (3.39) | 4.94 (2.17) | 5.46 (2.38) |  | 2.16 | 0.117 |
| Reappraisal | 5.17 (2.14) | 5.40 (2.23) | 5.11 (2.16) |  | 0.45 | 0.637 |
| Putting into perspective | 7.09 (2.08) | 7.56 (2.13) | 7.16 (2.10) |  | 1.27 | 0.283 |

**Exploratory Moderation: Brief Summary**

We conducted exploratory moderation analyses to examine whether trait attachment avoidance and anxiety (*Ws*) moderated the effect of attachment imagery (anxious vs. secure [*D*_1_] and avoidant vs. secure [*D*_2_]) on: (a) state hyperactivating ER, (b) state deactivating ER, (c) state paranoia, and (c) state anxiety. However, there were no moderation effects on any of these outcomes suggesting that participants’ dispositional attachment style did not interact with the impact of the imagery primes
